# Curriculum Innovation in Times of the COVID-19 Pandemic: The Thinking-Based Instruction Theory and Its Application

**DOI:** 10.3389/fpsyg.2021.601607

**Published:** 2021-04-12

**Authors:** Yangping Li, Xinru Zhang, David Yun Dai, Weiping Hu

**Affiliations:** ^1^Key Laboratory of Modern Teaching Technology (Ministry of Education), Shaanxi Normal University, Xi’an, China; ^2^Department of Psychology, Pennsylvania State University, University Park, PA, United States; ^3^Department of Educational and Counseling Psychology, State University of New York at Albany, Albany, NY, United States; ^4^Shaanxi Normal University Branch, Collaborative Innovation Center of Assessment Toward Basic Education Quality at Beijing Normal University, Xi’an, China

**Keywords:** curriculum innovation, thinking-based instruction theory, learning at home, key competencies, online learning

## Abstract

At the beginning of 2020, to stop the spread of the coronavirus disease (COVID-19) to the campus, the Ministry of Education of China launched a policy “Suspension of classes without suspending schooling” for the spring semester of 2020. However, the drawbacks of online teaching (e.g., students’ inadequate autonomous learning, the lack of effective online instruction) forced us to modify teaching strategies during this special period, especially developing courses that are suitable for student learning at home and improving their key competencies. In order to solve these problems, this study introduces some theoretical exploration and practical work of curriculum design under the guidance of thinking-based instruction theory (TBIT) during the pandemic. We firstly introduce TBIT, and elaborate on the curriculum design under the TBIT theoretical frame. Then we describe a series of TBIT-based micro-courses with the pandemic as background. A descriptive study is reported to illustrate the effects of three micro-courses. Results showed that, compared to national curricula, the TBIT-based micro-courses not only improved the course quality but also enhanced students’ motivation and facilitated their online learning behavior (such as interactive communication) for the online courses. The current study has important implications for how to design effective and interesting online courses suitable under pandemic and capable of improving students’ thinking abilities and key competencies.

## Introduction

The novel coronavirus pneumonia (COVID-19) broke out at the end of 2019 and has spread out quickly throughout the world. This major public health emergency led to a dramatic change all over the world, affecting all aspects of social life, including education (Naciri et al., 2020; Sintema, 2020a,b; Van Lancker and Parolin, 2020). In order to stop the spread of COVID-19 to campus and ensuring the safety and health of teachers and students, most of the countries have changed their teaching methods from face-to-face to online. The same goes for China, the Ministry of Education of China (MOE) has launched an emergency policy called “Suspension of classes without suspending schooling (SCWSS)” for the spring semester of 2020 and let students start online learning at home (Ministry of Education of the People’s Republic of China, 2020a).

This unprecedented policy provided a new space for online learning, which helps learners through remote instruction (Kumar Basak et al., 2018). Though there are many advantages (e.g., flexible ways of learning, the higher degree of freedom for students, and better retention) of online learning (Radović-Marković, 2010; de Oliveira et al., 2018; Srivastava, 2019; Wong, 2020), its drawbacks cannot be ignored, especially in two aspects: autonomous learning ability at home for students and the quality of teaching online for teachers. For examples, students who engage in autonomous learning at home may experience a lack of motivation (Martin and Bolliger, 2018; Li et al., 2020; Ouyang et al., 2020) and a lack of adequate planning, monitoring, and reflection (Arkorful and Abaidoo, 2015; Durksen et al., 2016; Wong, 2020; Zhou et al., 2020). In regards to teaching, teachers may lack proper theoretical guidance in online teaching (especially a lack of guidance for cultivating key competencies in online classes; Hu, 2017), or may experience the lack of sufficient interactive communication between teachers and students (de Oliveira et al., 2018; Alshamrani, 2019; Srivastava, 2019; Bao, 2020).

Additionally, with regards to teaching contents, the new policy (SCWSS) stipulated that the online classes in this special period should reflect learning in a broad sense, which not only referred to the structured learning of the national curricula (i.e., Chinese, Math, English, etc.) but should cover the learning of a wide range of content. For instance, the knowledge of pandemic prevention and control should be disseminated, and the life education classes, public safety education, and mental health education should be included (Ministry of Education of the People’s Republic of China, 2020b).

Under the circumstances, recent curriculum reform and many policy documents might give us directions to guide the curriculum innovation during COVID-19. For example, the Organization for Economic Co-operation and Development (OECD) emphasizes the important role of key competencies in student development (OECD, 2018, 2019), and the OECD Education 2030 project suggests that “creating new value” and “reconciling tensions and dilemmas” are two important categories of competencies for future-ready students (OECD, 2018). Creating new value mainly refers to cultivating the creative thinking, while reconciling tensions and dilemmas refers to the ability to apply knowledge of both broad and specialized kinds, to meet complex demands beyond just acquisition of knowledge; it requires students to master a wide range of disciplinary and interdisciplinary knowledge and think and act in a more integrated way to solve problems. In this case, the popular “Maker” movement and science, technology, engineering, and mathematics (STEM) education have made great strides (Bevan et al., 2015; Martín-Páez et al., 2019). Meanwhile, two other promising strategies are important in curricular innovation—using “big ideas” to define key concepts and applying “learning progression (LP).” “Big ideas” was proposed by the American Association for the Advancement of Science’s Project 2061 (AAAS, 2005). The integrated course content around big ideas contributes to establishing a systematic knowledge structure in students’ minds (Lelliott and Rollnick, 2009; Duncan and Rivet, 2013; Plummer et al., 2015). LP means that the teaching of a concept should be carried out in sequence due to the gradual and even complex nature of building mental apparatus (Hu, 2019). These two strategies have been emphasized in a new round of science curriculum reform in China since 2017 (Yao and Guo, 2018). Curriculum innovations are made in various countries along the same line to varying degrees (Chugh et al., 2017; Tan et al., 2017; Cunningham et al., 2019; Brown and Livstrom, 2020; Lemay and Moreau, 2020).

Taken together, under the pandemic situation, it has become an urgent matter to figure out how to design courses that, under the guidance of an effective instruction theory, could arouse students motivation, suitable for students to study at home, and meet the requirements of curriculum reform (i.e., conducive to students’ intellectual development, while enabling students to cultivate their key competencies, and facilitating systematic knowledge construction).

### The Present Study

In order to address the problem mentioned above, this study aims to introduce a widely used instruction theory—TBIT, especially how it is used in course design and curriculum implementation in the special time of the pandemic. To demonstrate how it works, we will delineate three TBIT-based micro-courses. Additionally, the effectiveness of these courses will be examined on three important aspects of online courses: the course quality, learning experience, and online learning behaviors.

#### Thinking-Based Instruction Theory (TBIT)

Based on Hu’s thinking ability structure model (TASM, Hu et al., 2011), and constructivist theories of learning, such as Piaget’s cognitive development theory (see Evans, 1973), and Vygotsky’s social constructivist theory (Vygotsky, 1978), Hu et al. (2011) developed the thinking-based introduction theory (TBIT) in the aim of cultivating students’ thinking abilities through instructional activities. The most prominent feature of TBIT is that it proposes that thinking activity plays a key role in the learning process. It holds that focusing teaching efforts on the cultivation of thinking ability can promote the development of students and the cultivation of thinking ability could serve as the starting point of the cultivation of any subject or interdisciplinary content. Meanwhile, this theory proposes five principles and six instructional steps to guide the courses design and instructional activities (Lin and Hu, 2010; Hu et al., 2011; Hu, 2015).

TASM is regarded as a theoretical foundation on which TBIT was built. This theory abstracts thinking ability into a three-dimensional structure. TASM suggests that thinking content is used as the carrier, the thinking methods are the main thread, and thinking qualities are taught in each activity. They all include several different elements (Please see the figure of the model in Hu et al., 2011):

(a) Thinking content: mathematics, language and literature, science, society, art, other disciplines, and daily life experiences.
(b) Thinking methods: e.g., space cognition, comparison, classification, inductive and deductive reasoning, reorganization, brainstorming, transfer, questioning.
(c) Thinking quality: profundity, flexibility, critical thinking, agility, and originality.

This model suggests that the cultivation of thinking ability requires the teaching of thinking methods, the training of thinking quality, and all the training must be set within the context of a body of knowledge.

What distinguishes TBIT from the previous constructivist theories are two points: active thinking and specific guidance of the instructional procedure. On the one hand, based on the TASM, TBIT proposes that thinking activity plays a key role in the learning process, and suggests that the development of habits of mind entails teaching students to successfully use the different thinking methods with various contents and to ensure the quality of thinking. On the other hand, TBIT provides a prescribed, detailed sequence of activities to cultivate active thinking. Specifically, TBIT specifies what teachers should do to achieve the goal of improving students’ thinking ability. Hu proposes five teaching principles and six basic instructional steps when designing and implementing courses (Hu and Wei, 2010; Lin and Hu, 2010). These principles and instructional steps are based on years of teaching experience and involve sequenced activities and curriculum procedures. The five principles are (1) stimulating interest and motivation; (2) cognitive conflict; (3) knowledge-construction; (4) self-regulation and metacognition; (5) application and transfer. The six steps are based on the five principles and guide the curriculum design and instructional activities. The six steps are (1) situation creation; (2) questioning; (3) independent inquiry; (4) cooperation and communication; (5) summary and reflection; (6) application and transfer. The detailed explanations of these five principles and the six steps will be introduced in the curriculum implementation section of this paper.

Many attempts have been made in curriculum development based on TBIT, and the effectiveness of this theory has been well-established for many years. Around 2005, Hu started to conduct classroom teaching practice based on TBIT. From then on, the instructional principles are formulated gradually. Up to 2015, the TBIT curriculum as a model of curriculum innovation had been implemented in 300 primary and secondary schools, and more than 200,000 students took part in research on TBIT (Hu, 2015). As of now, our record indicates that this intervention program has been implemented in more than 2,000 primary and secondary schools. For example, the Learn to Think (LTT) curriculum is a typical curriculum developed based on TBIT (Hu et al., 2011, 2013). In two 4-year longitudinal studies, the LTT curriculum showed significant effects on improving students’ thinking skills and motivation (Hu et al., 2016), and academic performance (Hu et al., 2011). Subsequent studies also evidenced its effect on improving thinking ability and creativity in preschoolers (Bai et al., 2020), primary school students (Hu et al., 2016), and secondary school students (Hu et al., 2013).

#### TBIT-Based Courses Design

**FIGURE 1:**
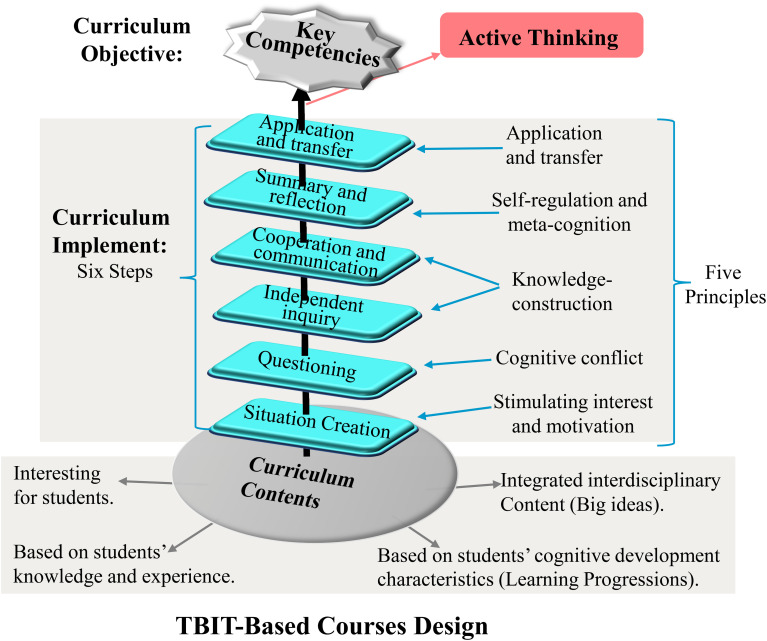
The TBIT-based courses design.

In this paper, based on TBIT-related experience accumulated and applied in the course practice over the years, the policy requirements, and the curriculum reform requirements, we detailed describe the TBIT-based course design method in guiding the course design (as shown in Figure 1). Specifically, we stress three critical elements (curriculum objective, curriculum implement, and curriculum contents) of course design. Firstly, the curriculum objectives should be focused on cultivating key competencies of students. Secondly, the curriculum contents should emphasize four points: (a) stimulating students’ interest, (b) teaching around big ideas and integrated interdisciplinary content, (c) focusing on cultivating students’ key competencies and facilitating their positive thinking, and (d) choosing the course contents based on students’ cognitive development characteristics. Thirdly, the curriculum implementation should be consistent with the five principles and the six steps of the TBIT.

##### Curriculum Objectives

Curriculum objectives here refer to the specific goal of a course, in which, educational effects are expected to be achieved through the curriculum (Liu, 2004). They play the role of the intermediate link between macroscopic education vision and concrete education and teaching practice. Typically, curriculum objectives not only determine the direction of curriculum development but also contribute to the selection and organization of curriculum content as well as the implementation of the curriculum.

According to the requirements of recent education documents from international organizations (i.e., OECD) and other countries (e.g., the United States, Singapore, Finland, etc.), as well as the new round of curriculum reform in China, they all emphasize that curriculum objectives should mainly focus on key competencies (Ananiadou and Claro, 2009; Singapore, 2010; Vahtivuori-Hänninen et al., 2014; OECD, 2018, 2019; Yao and Guo, 2018).

Specifically, key competency refers to the necessary characters and key abilities that students should possess to adapt to the needs of lifelong development and social development (OECD, 2018, 2019), which is particularly important to help students better face the promises and challenges of the twenty-first century. However, curricula in China usually place a premium on knowledge and skills, which is deemed insufficient to support the well-rounded development of students (Cui, 2016; Hu, 2016). To face this issue, the Chinese government has proposed six basic qualities of key competency—humanistic background, scientific spirit, learning, healthy life, responsibility, and practice and innovation, that students should be cultivated in school (Lin, 2016a,b). Meanwhile, consistent with the direction of international curriculum reform and the requirement of government documents, TBIT emphasizes that curriculum objectives should focus on cultivating the key competencies (Hu et al., 2013; Hu, 2015), and the competency-based curriculum should help students to achieve a deep understanding of knowledge as well as applicate knowledge flexibly in real situations, to improve their cooperation ability and communication ability, and to enhance their intrinsic learning motivation and autonomous learning ability (Hu, 2017, 2019; Cunningham et al., 2019).

To cultivating the key competencies in classroom practice, TBIT propose that the core of cultivating competencies is cultivating students’ higher-order thinking (i.e., critical thinking and creative thinking; Hu et al., 2013; Hu, 2019). The process of knowledge internalization and scientific inquiry both require higher-order thinking (Resnick, 1987; Richland and Simms, 2015). Applying knowledge in solving complex problems, conducting innovative activities in everyday life, and critical thinking all rely on higher-order thinking (Shou, 2018; Hu, 2019). Therefore, training higher-order thinking is the necessary part of cultivating key competencies.

In terms of the specific thinking training, based on TASM (Hu and Luo, 2001; Hu and Lin, 2003), in which thinking ability is a three-component structure, composed of the content, method, and quality of thinking. Teachers can train students to learn to use various thinking methods and improve various aspects of thinking quality according to thinking content in colorful course activities.

##### Curriculum Contents

The curriculum content is design to achieve the curriculum goal. Curriculum content refers to what a course should contain, and how the learning materials should be organized. When designing a course, according to TBIT, four points should be emphasized: (a) stimulating students’ interest, (b) teaching around big ideas and integrated interdisciplinary content, (c) focusing on cultivating students’ key competencies and facilitating their active thinking, and (d) choosing the course contents based on students’ cognitive development characteristics.

As to the first point, the materials the teachers chose to teach should first enable to stimulate students’ interest, especially in online learning, because lacking autonomous learning ability is the most severe problem in online learning (Liang et al., 2020; Shang et al., 2020). Meanwhile, motivation was found positively related to persistence (Moreira et al., 2013; Liao et al., 2014) and could significantly predict students’ academic performance in certain subjects and creativity (Lee et al., 2016; Salta and Koulougliotis, 2020; Wang et al., 2021). How do we stimulate students’ interest? According to TBIT (Hu et al., 2011), teachers should choose materials that are appropriate and based on students’ prior knowledge, experience, and interests for their ages. Therefore, the first principle of the five teaching principles in TBIT is “stimulating interest and motivation,” which means to use content that students are familiar with to set the situations, so as to attract students’ interest and improve their motivation. For example, most of the courses in this study we designed have chosen content related to pandemic to attract students, which is familiar to students in this special period and can capture their attention more easily. Additionally, regarding the course content, due to the abilities are domain-related, the course contents we chose were also diversified, which cover almost all the domains, such as Chinese, math, English, science, arts, health, society, and life skills. This is also consistent with the content requirements of the new policy (SCWSS) of the ministry of education.

Secondly, the teaching contents focus on big ideas and integrated interdisciplinary contents, which are the basis for TBIT-based micro-courses. The big ideas are the center of a knowledge field, and it is the conceptual knowledge that students need to understand and then later apply (Lelliott and Rollnick, 2009). Notably, the integrated course content around big ideas contributes to establishing a systematic knowledge structure in students’ minds (Duncan and Rivet, 2013). Furthermore, teachers should design interdisciplinary contents or activities, which are more attractive to students and conducive to their flexible thinking. Meanwhile, it could facilitate their abilities to relate various information to the semantic networks in their mind, which could, in turn, enrich their knowledge network. Also, this interdisciplinary design can facilitate students’ abilities of creativity and transfer, as highly creative people are found to have a more interactive, flexible, and clustered semantic network compared to less creative people (Kenett et al., 2014; Benedek et al., 2017; Li et al., 2021).

Thirdly and most importantly, the course contents should focus on cultivating students’ key competencies and facilitating their thinking. The training of key competencies is the core of the recent curriculum reform in China (Lin, 2016a,b; Yao and Guo, 2018), which is also the main aim of our courses. Therefore, every course in our curriculum should focus on cultivating students’ multiple key competencies. Meanwhile, we set up some teaching steps to activate students to active thinking so as to enhance the flexibility, creativity, criticalness, agility, and depth of their thinking, and eventually help them build the corresponding key competencies through knowledge building and thinking training. For example, the “cognitive conflict” and “summarize and reflection” steps based on TBIT in our courses aim at provoking students’ deep thinking about the problems they have encountered in a course; later in the course, their metacognitive skills could be improved through the summary and reflection phase.

In the end, the course content design should be based on students’ cognitive development characteristics (Jin et al., 2019; Sikorski, 2019). Vygotsky (1978) proposed that teaching should help students reach the zone of proximal development in each phase (Vygotsky, 1978). Therefore, we need the “learning progressions (LP)” design, which means the development of student thinking is coherent and gradual (Hu, 2019). When they study or research a certain concept or topic, their ways of thinking are carried out in sequence. Essentially, learning progression is the in-depth and continuous understanding of core concepts (Alonzo and Steedle, 2009). This kind of design can let teachers help students reach the zone of proximal development in each phase (Vygotsky, 1978), thus achieving effective teaching. At the same time, the development of students’ understanding of core concepts through progressive learning will help students form a solid knowledge structure, understand scientific concepts in-depth, and improve their ability to solve problems. This LP design also has become the core of science curriculum reform in contemporary basic education (Duncan and Rivet, 2013) and has been verified effective in various subjects, such as physics (Herrmann-Abell and DeBoer, 2018) and astronomy (Plummer et al., 2015). Therefore, in our courses, we designed content at suitable difficulty levels for students in different grades, and appropriate for their ages and cognitive developmental characteristics.

##### Curriculum Implementation

Curriculum implementation is the main process to achieve the curriculum objectives. TBIT could give us effective guidance in curriculum implementation. TBIT takes active thinking as core and proposes five principles and six steps to conduct effective teaching (Zheng and Wang, 2015; Hu et al., 2016; Xie and Xu, 2019; Bai et al., 2020). We will give a detailed explanation for the five principles firstly.

(1) *Stimulating interest and motivation.* A good course should first attract students’ attention. So, the course should stimulate student’s interest and motivation firstly. Teachers should choose materials, contents, and stories that students are interested in as warming-up. The motivation was found positively relating to students’ academic performance (Lee et al., 2016; Salta and Koulougliotis, 2020) and creativity (Wang et al., 2021).
(2) *Cognitive conflict.* Cognitive conflict is used to describe a situation that students feel confused or puzzled, especially when that is inconsistent with their previous experience or understanding (Kang et al., 2004). Rooted in Piaget’s notion of disequilibrium, which drives cognitive effort to resolve discrepancies, cognitive conflict was found helpful in improving students’ motivation and arousing their curiosity about the course contents (Kang et al., 2010; Bao et al., 2014).
(3) *Knowledge construction.* Knowledge construction includes cognitive construction and social construction. Cognitive construction is a process in which learners actively construct their internal mental representations, which are realized through the interaction of old and new experiences (Evans, 1973). Social construction is based on Vygotsky’s viewpoint that social interaction is central to children’s cognitive development (Vygotsky, 1978). Social construction here involves teacher–student interactions and student–student interactions. Teachers should design courses with discussion sessions, in which students could argue and share their views with other students. In this process, knowledge is socially constructed, and specific ways of thinking are mutually reinforced. As previous research found, compared to traditional instruction, courses that involve collaborative experimentation and interactions, or storytelling, hands-on experiments, and drama, could improve preschoolers’ thinking ability (Kakana et al., 2009). Also, cooperative learning and peer-learning partnership could increase students’ motivation (Eisenkopf, 2010).
(4) *Metacognition and self-regulation.* Metacognition and self-regulation are the basis of all of these thinking methods. Metacognition is the awareness and control of one’s thinking processes (Brown, 1987; Jang et al., 2020). Self-regulation refers to learners’ drive by the motivation or use strategies to initiate and sustain focused goal-directed activities while ignoring distractions or setbacks (Schunk et al., 2014). A good course should guide students to improve their meta-cognitive ability to find what strategies they have used when solving problems, and teachers should help students learn to monitor their thinking, internalize the monitoring process, and make the monitoring process as part of their habitual mode of thinking (Hu et al., 2016).
(5) *Application and transfer.* Given that the instructional process is based on the specific course contents or activities, we would like to develop students’ ability to draw inferences from one instance or apply what they have learned from courses to the problems in real life. Thus, a good course should involve the application and transfer component, which could not only facilitate students’ transfer ability but also help students gain self-efficacy when they make a successful transfer, which in turn improves their motivation to apply what they have learned in other subjects (Iswahyudi et al., 2019).

Based on these five principles, which constitute the core of active thinking, Hu et al. (2016) proposed six steps to guide instructional planning, which teachers can implement in their design of specific activities to arouse and cultivate students’ active thinking. The six steps are as follows:

(1) Situation creation. This step is based on the first principle “Stimulating interest and motivation”. At the beginning of a course, the teacher should choose the contents that students are interested in to create an interesting situation to attract their attention. For example, in the course “A conversation between the virus and me” developed by Jiuhua Heping Primary School, teachers take the spreading of COVID-19 as the background to attract student’s attention.
(2) Questioning. This step is based on the principle “cognitive conflict” and is meant to arouse students’ deep thinking about the theme. For example, in biology lessons, to guide students to explore the structure and characteristics of the organism that causes Covid-19, the teacher should lead the students to ask questions about the topic, such as “Is COVID-19 a form of life? Does it grow?”
(3) Independent inquiry. This step is based on the principle “knowledge construction”. This step could help students get involved in the cognitive construction process, and improve the quality of the discussion. This process encourages students to relate their previous knowledge and experiences with the new questions, and thus gain new insights into the new situation (Baanqud et al., 2020). The previous study found that a group can think more creatively if individual members think independently before group discussion (Chen et al., 2021).
(4) Cooperation and communication. This step is also based on the principle “knowledge construction” and it belongs to social construction. Teachers should set up discussion sessions to facilitate social construction. For example, during the pandemic, teachers encourage students to share the questions that they have in mind about the virus or the methods to protect themselves from the virus on the online chat platform.
(5) Summary and reflection. This step is based on the principle “self-regulation and metacognition”. Here, teachers should guide students to reflect on and summarize the thinking methods, thinking strategies, problem-finding, problem-solving methods, and what they have learned from the activities.
(6) Application and transfer. This step is based on the principle “application and transfer”. Teachers should guide students to actively use the strategies and methods they have learned in other domains. The transfer of knowledge truly indicates that cognitive representations are transformed into abilities and competencies.

### The Three Courses Designed Under the Guidance of TBIT

Under the guidance of TBIT (Hu et al., 2011), and considering the requirements of the SCWSS policy and the curriculum reform contents, teachers in three primary schools designed three courses with COVID-19 as background. They are Home + X course (designed by teachers in Jiuhua Heping primary school in Hunan), 551-course (designed by teachers in Yuxin School affiliated to Capital Normal University in Beijing), Flower-centered course (designed by teachers in Liwan primary school in Guangzhou). The introduction of these three courses is as follows.

#### Home + X Course

Jiuhua Heping Primary School in Xiangtan City, Hunan Province is one of the national experimental schools for TBIT, and it pays lots of attention to cultivating students’ thinking ability in daily classroom teaching. In the context of the pandemic, the school, based on TBIT, infiltrated a variety of thinking methods, integrated interdisciplinary contents related to the pandemic in various teaching activities, and independently developed a set of “Home + X” courses that integrate fun, depth, and implications. This course aims to develop students’ cognitive understanding, inquiry skills, thinking, and other comprehensive abilities during the pandemic period, as part of the cultivation of students’ key competencies.

The specific meaning of “Home + X” is as follows. “Home” means a package that includes multi-faceted integration of contents, such as multi-disciplinary integration, multi-activity interconnection, multiple participants, and multi-dimensional goals and evaluations. “+” expresses the series under the big themes, such as the “thinking challenge” series to develop thinking skills, the “interactive time” series to build a display platform, the “sharing and evaluation” series to encourage creative comments, and the “anti-pandemic methods” series focusing on life education. “X” represents the small activities under the big themes. For example, all subjects and classes can carry out specific activities (involve characteristics of school and class) under the guiding principle of multiple integrations. In short, the “Home + X” course is presented in the form of a diversified integration of major themes with a series of small activities, to help students to relieve panic and anxiety in enjoyable activities, and turn the period of passive absorption at home into an opportunity for proactive learning and growth.

**FIGURE 2:**
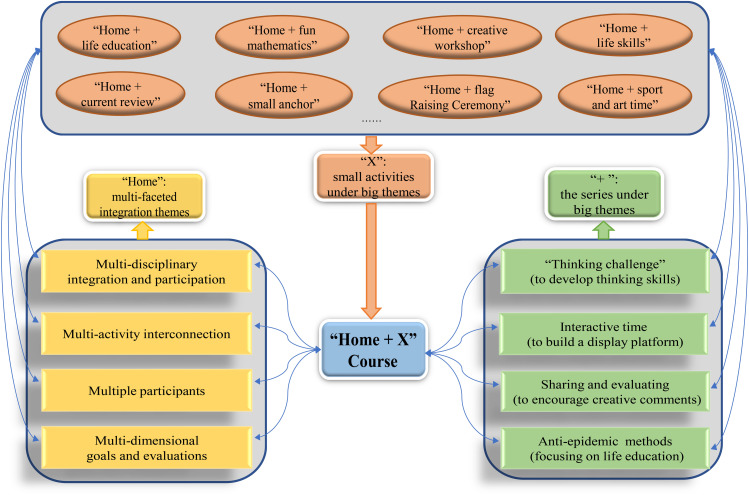
The framework of the “Home + X” course.

In terms of the content of the course, based on the characteristics of different disciplines as they have a bearing on the pandemic, teachers in this school designed a series of theme-based activities, including “home + life education,” “home + current review,” “home + fun mathematics,” “home + small anchor,” “home + creative workshop,” “home + life skills,” “home + sport and art time,” and so on. The framework of the course is shown in Figure 2.

An example of “Home + X” courses: “Home + life education.”

“Home + life education” is part of the series of “anti-pandemic methods.” The teachers in this school organized lots of valuable content to record many micro-classes, such as collecting information about the new coronavirus and make full use of popular science materials generated by the pandemic. In this way, students are guided to actively pay attention to the progress of the pandemic, understand implications of wildlife protection, learn about virus prevention and control, etc., to help students tackle the unknown, eliminate panic, and learn to protect themselves. This activity also suggests ways in which students can influence their family members through their actions and make them activists for pandemic prevention. The specific activities arrangement is shown in Table 1.

**TABLE 1:** “Home + life education” theme series activities arrangement.

**Time**	**Activity name**	**Content**	**Activity ways**	**Integrable subjects**	**Fusion series**
The 1st Week	I am a family epidemic prevention supervisor.	Learn about epidemic; learn to wear masks; be a good family epidemic prevention activist.	Video, micro-classes, etc.	Science, biology.	Sharing and evaluating; anti-epidemic methods.
The 2nd Week	I am a little guardian of family health.	Temperature test, to be a good supervisor and analyst (Figure 3 shows one figure made by a student)	hands-on practice, recording	Science, mathematics.	Sharing and evaluating; methods of anti-epidemic.
The 3rd Week	A conversation between me and the virus.	Pay attention to information about epidemic and virus, watch the reproduction and spread of viruses and bacteria, and then carry out an imaginative conversation with the virus.	Watching, recording, and expressing.	Science and literacy.	Thinking challenge; sharing and evaluation.
The 4th Week	I am a campus prevention and control organizer.	Design and prepare for the start of school, design the prevention and control icons, and the prevention and control plans for individuals and class.	Hands record, class.	Language, art.	Thinking challenge, sharing and evaluating

**FIGURE 3:**
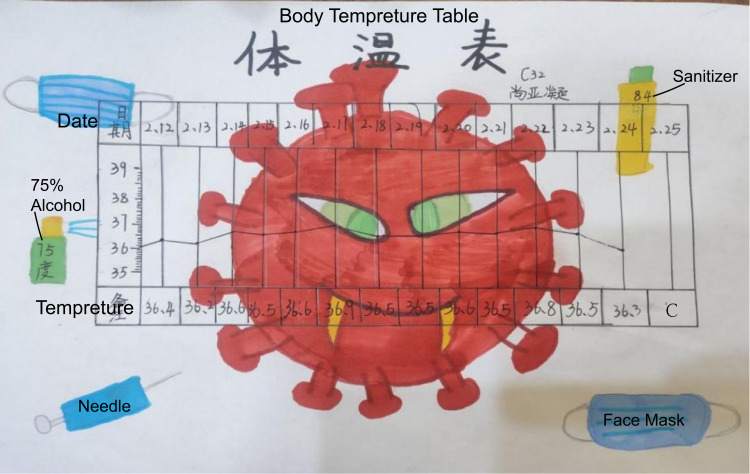
Polyline statistical of body temperature recorded by one student.

In this activity, teachers set up a series of activities to make students pay more attention to social issues and life around them as relevant to the pandemic to turn simple notification and warning into preventive practice and truly help students translate the knowledge on the books into real-life practice. At the same time, the activities help students analyze and solve problems scientifically and logically, and establish scientific concepts and cultivate scientific thinking. Following is a body temperature recording sheet designed by a student (Figure 3).

#### 551-Course

Thinking-based instruction theory provides strong theoretical support for the school’s curriculum reform and points out the future development direction. After continuous exploration, the Yuxin school in Beijing constructed the “551” thinking classroom teaching model based on TBIT. The connotation of “551” is “one core, five principles, and five steps.”

Firstly, “one core” refers to the aim of the course design—improving students’ thinking ability, which is the same as the core of TBIT. Secondly, “Five principles” refers to the basic tenets of instructional design or the five basic principles of thinking-based teaching: stimulating interest and motivation, cognitive conflict, knowledge-construction, metacognition, and application transfer. Based on these five basic principles, this courses put forward five concepts of instructional design: (1) Orderly (clear classroom steps and leading the rhythm of thinking); (2) Interesting (set thinking points and stimulate students’ desire for knowledge); (3) Effective (focus on problem teaching and strengthen thinking training); (4) Emotional (enhancing teacher-student interaction and improving thinking quality); (5) Useful (extending the thinking path and linking reality themes). Thirdly, “Five steps” adapted from the six teaching steps of teaching design, which were adapted from the six basic teaching steps advocated by TBIT (create situations, questioning, independent inquiry, cooperation and communication, summary and reflection, and application and transfer). The specific meaning of “five steps” are (1) Situation creation and inspiring thinking; (2) Exploring independently, and thinking quietly; (3) Teacher-student cooperation, dialectical thinking; (4) Practice implementation, feedback, and thinking; (5) Returning to life scenes (situations) and expanding thinking. Meanwhile, the teachers expanded the meaning of these five steps in the context of the coronavirus pandemic.

According to the nature of “learning progressions” in the course content design (Alonzo and Steedle, 2009), each subject of the course is designed according to students’ cognitive development rules. For example, the science group has set curriculum goals for junior, middle, and senior students separately. The course for junior students put increased emphasis on arousing students’ curiosity in science and stimulating children’s interest through scientific experiments and short videos. In scientific experiments, we focus on cultivating children’s observation and hands-on practical abilities, and guiding children to think about problems in a scientific way. The course in middle grades aims to arouse students’ interest in hands-on activities through science games, small experiments, and short videos. Starting from simple scientific experiments, it helps students establish a scientific way of thinking that uses data to verify conjectures, gradually improves students’ experimental operation skills, and establishes conceptual foundations for scientific experiments. In the upper grades, students are encouraged to design experiments by themselves and learn to explain the results of the experiments using experimental logic. This stage focuses on cultivating students’ ability to collect and organize evidence independently as well as critically examine and learn from the collected data, and eventually draw scientific conclusions.

In terms of the content of a course, each subject of the course is based on the principles of big ideas, learning progressions, and key competencies which were emphasized in the content design of the course (Lin, 2016a,b; Hu, 2019). At the same time, in the context of the pandemic, different subjects drew their themes. For example, the subjects of Chinese and English were mainly taught with traditional recitation, picture book reading, and connections to the pandemic. An example reads like the following: Please read the picture book of fighting the pandemic, then think about the questions following: ① Why did this pandemic break out? ② What is a coronavirus? ③ What should we pay attention to during the pandemic? ④ What are the touching stories that impressed you most during this pandemic? ⑤ What changes do you think we need to make in the future? Additionally, art disciplines such as fine arts and music mainly focus on creative activity with current events. For example, the art discipline revolves around the themes of “my city and me,” “my country and me,” “my world and me,” and “my earth and me,” generating narratives of students’ own life and social focus events with artistic creation. In short, in a series of courses, through videos and teacher demonstrations, students are guided to pay attention to life, express feelings about life, think about life, and practice art with life, to cultivate students’ sensitivities to language, health, and art, and other aspects of personal life and growth, as well as improve students’ analytic and reasoning skills, and creative and critical thinking.

Besides, the school pays close attention to the mental health of students, and promptly follows up and understands students with emotional disturbances. Students who have difficulties with their families, especially those who are indeed neglected by parents in the front line of pandemic prevention and control, were provided with necessary services.

#### Flower-Centered Course

The Liwan primary school in Guangdong province is a new school established in 2019. Guangzhou is known as the “City of Flowers,” also “Flower” is a characteristic culture of Guangzhou. During the pandemic, based on the connotation of TBIT, the president of this school asked teachers to integrate Guangzhou’s characteristic “flower” culture into the curriculum, and infiltrating various thinking methods and subject-based key competencies. Finally, the teachers developed the “Colorful World” series of courses through the creation of flower-related situations in the general subject teaching, which greatly triggered students’ enthusiasm for learning.

In terms of principles of curriculum design, the entire curriculum revolves around the theme of “flowers” and includes three dimensions: human and nature, human and self, and human and society. Different courses are associated with different dimensions according to their respective topics. Secondly, based on the interdisciplinary design concept, all courses, to varying degrees, integrate the knowledge of Chinese, mathematics, English, ethics, the rule of law, sports, music, art, science, psychology, dance, health, and other subjects. This kind of design breaks the boundaries of disciplines and facilitates multi-disciplinary and interdisciplinary teaching. It aims to help students break mental sets, improve their ability to solve problems, and at the same time promote students’ overall development. Meantime, in this curriculum, multiple thinking elements (i.e., purpose, viewpoint, problem, hypothesis, concept, information, inference, result, and meaning) are connected, spiraling upward, and are flexibly reflected in the design of integrated contents.

According to the requirements of curriculum design, in terms of progression design (Hu, 2019), the whole set of curriculum design is progressive, guiding children from knowledge acquisition to thinking and creation. To facilitate the implementation of the course, each course is accompanied by a corresponding guiding plan for different learning needs. Students are asked to preview it before class, follow the plan in class and expand the contents after class to improve the quality of learning.

One course example is like the following: “Hundred Flowers blossom in Spring, Knowing Flowers in a Flower City.” This course introduces students to the characteristics of many kinds of flowers and guides them to learn the magical uses of flowers. At the same time, popularize the edible and medicinal value of flowers could help students to know how to use flowers for self-health care in their lives. This course integrates the two dimensions of “human and nature” and “human and self,” as well as subjects of language and health. Meanwhile, this course cultivates students’ information collection ability, thinking ability, and expression ability.

### Hypothesises

In order to design courses suitable for home-based study, and to meet the requirements of curriculum reform, this study has introduced the TBIT theory. According to this theory, we gave the specific guidance of the three critical elements of curriculum design (curriculum objectives, curriculum contents, and curriculum implementation), and provided three TBIT-based micro-courses. Meanwhile, we tested the instructional effects of the TBIT-based micro-courses compare to the national curricula (e.g., Chinese and math) on three aspects: students’ evaluation of the quality of the courses, learning experience, and online learning behaviors. Regarding the courses, the TBIT-based micro-courses and the traditional curricula are both conducted to facilitate students’ learning and development. The national curricula were conducted to help students acquire basic, systematic, and in-depth knowledge of each subject, while TBIT-based micro-courses mainly focus on improving students’ core competencies by engaging their active thinking.

Since the design of the three TBIT-based micro-courses was based on theoretical guidance, we expect that students’ evaluation of the TBIT-based micro-courses to be better than the national curricula (Hypothesis 1). Given the courses that we designed under TBIT before (i.e., the learn to think course, LTT) has improved students’ motivation (Hu et al., 2016), we expect that the TBIT-based micro-courses are more effective than the national curricula in stimulating students’ learning motivation (Hypothesis 2). Besides, since the courses have a strong emphasis on online interaction, we expect that students’ online study behaviors (i.e., interactive communication, learning attitude, and persistence) in the TBIT-based micro-courses are better than those in the national curricula (Hypothesis 3).

## Materials and Methods

### Participants

Both students and teachers participated in a feedback survey on the course. Among them, questionnaires were used to collect quantitative data for students. Besides, informal interviews were conducted with teachers to collect qualitative data to further corroborate with the assessment results. The study protocol was approved by the local ethics committee. All participants provided informed consent before the data collection process.

In each of the three schools, students from one class were randomly selected to participate in the survey around the thinking-based courses, and students from another class were randomly selected to participate in the survey around the national curricula. The original sample is 262. Forty-eight participants were excluded because they skipped a few questions of the questionnaires. So, the final sample size for the present analysis was 214 participants (102 females, see demographic information in Table 2). Participants at the three schools were at different grades. Considering that older students have stronger metacognitive abilities and should give deeper and more representative feedback on the curriculum, participants at Yuxin School and Jiuhua Heping School were selected from fifth grade (*M*_age_ = 11.04, *SD* = 0.55). Since Liwan School is a newly built school with only first-year students, the participants there are younger (*M*_age_ = 7.02, *SD* = 0.65). The students in the two classes in each school were matched in terms of students’ academic performance and family backgrounds.

**TABLE 2:** Descriptive statistics of subjects in two assessments and three schools.

**Assessment**	**Variables**	**Jiuhua**		
			**Yuxin School**	**Liwan School**
**objective**		**Heping School**		
**TBIT-bases micro-courses**		**Home + X course**	**551-course**	**Flower-centered course**
	Age (M ± SD)	11.20 (0.50)	10.89 (0.61)	7.00 (0.48)
	Number	53	37	31
**National curricula**				
	Age (M ± SD)	11.13 (0.57)	10.95 (0.51)	7.04 (0.75)
	Number	30	39	24

Fourteen teachers in the three schools (4–6 in each) participated in the interview toward their perspective on the TBIT-based micro-courses. These teachers have been engaged in the curriculum design and teaching of the TBIT-based micro-courses, and the subjects they teach are diverse.

### Measurements

#### Course Experience Questionnaire

Students’ perceptions of the quality of their courses were measured by the Course Experience Questionnaire (CEQ) (Eley, 1998). It was originally designed by Ramsden (1991) and was adapted into a simplified version of 23 questions by Eley (1998) and has been widely used to evaluate teaching quality at the curriculum level (Byrne and Flood, 2003). CEQ consists of five subscales: good teaching, clear goals, appropriate workload, appropriate assessment, and generic skills. Considering our purpose of examining the quality of the course, only the last four dimensions were adopted (the “good teaching” part was excluded), with a total of 17 questions (α = 0.86). Students were asked to respond on a five-point Likert scale (1 = “strongly disagree,” 5 = “strongly agree”). Example items are “It was always easy to know the standard of work expected” and “The workload was too heavy.””.

#### Mayer Learning Experience Questionnaire

The Mayer Learning Experience Questionnaire (Moreno and Mayer, 2000; Stull et al., 2018) was used to measure students’ learning experience in terms of motivation, interest, understanding, the perceived difficulty of the material, and engagement. The scale contains eight items and was scored on a seven-point Likert scale (α = 0.87). An example item is “I enjoyed learning this way.”

#### Online Learning Behavior Questionnaire

Three subscales (i.e., interactive communication, persistence, and learning attitude) from the Online Learning Behavior Questionnaire (Li et al., 2013) were used to measure students’ online learning behaviors in traditional courses as well as thinking-based online micro-courses. The three subscales contain 12 items in total and were scored on a five-point Likert scale (α = 0.86). An example item is “I am willing to share my ideas with my classmates in the TBIT-based micro-courses (or ‘in the national curricula’).”

#### Informal Interviews Toward Teachers’ Perspectives on the Thinking-Based Courses

In addition to the questionnaires for students, informal interviews were conducted for teachers to qualitatively evaluate their perspectives toward the TBIT-based micro-courses. Four open-ended questions were used in the informal interviews: “Compared with previous courses, what are the features of the TBIT-based micro-courses during the pandemic?”, “How is the effect of TBIT-based micro-courses during the pandemic on improving students’ thinking ability?”, “How is the effect of TBIT-based micro-courses during the pandemic on improving students’ key competence?”, and “How about students’ engagement in learning TBIT-based micro-courses?”

### Procedure

The feedback survey was conducted in mid-June 2020. By the time the survey data were collected, students in each school had taken TBIT-based micro-courses at least 10 times and national curricula for more than 7 weeks (see Table 3). For the fifth-grade students, they received the link to the three questionnaires from their teachers. Students are required to use mobile phones or computers to open the link and complete the questionnaires. For the first-grade students, the printed questionnaire was handed out by the teachers, so the younger students could complete the questionnaire more easily.

**TABLE 3:** The class frequency and duration of the TBIT-based micro-courses and the national curricula by the time of data collection.

**Questions**	**Jiuhua Heping School**	**Yuxin School**	**Liwan School**	**Jiuhua Heping School**	**Yuxin School**	**Liwan School**
				
	**Home + X course**	**551-course**	**Flower-centered course**	**National curricula**		
How long have the students been taking this course (weeks)?	10	20	2	7	20	12
How many classes does the student attend per week?	1	2	10	14	20	25
How long does each class last (minutes)?	20–30	20	20	30	20	20

Besides the quantitative investigation, a qualitative interview was used to enrich data interpretation and enhance the validity of specific claims. Through this method, more comprehensive information will be collected, especially the information not investigated from the questionnaires. Regarding implementation, the day after the survey, the teachers in the three schools received a link for the informal interview. They were asked to anonymously answer four open-ended questions.

### Data Analysis

To assess the effects of the TBIT-based micro-courses, several independent *t*-tests (see Table 4) were conducted separately between the assessment on TBIT-based micro-courses and national curricula for three courses (Home + X course, 551-course, and Flower-centered course) on each aspect of the three questionnaires (CEQ, MLEQ, and OLBQ).

**TABLE 4:** Independent *t*-test results of two assessments on each dimension of the three questionnaires in three schools.

**Questionnaires and its aspects**	**Jiuhua Heping School**			**Yuxin School**			**Liwan School**		
	***t***	***df***	***p***	***t***	***df***	***p***	***t***	***df***	***p***
**Course Experience Questionnaire (CEQ)**									
Clear goals and standards	–0.96	81	0.342	1.80	74	0.076	2.42	53	0.019*
Appropriate workload	0.08	81	0.936	–1.10	74	0.274	–3.27	53	0.002**
Appropriate assessment	0.98	81	0.328	0.84	74	0.402	0.77	53	0.443
General skills	–0.99	81	0.326	1.13	74	0.262	1.39	53	0.169
**Mayer Learning Experience Questionnaire (MLEQ)**									
Motivation	2.10	81	0.038*	2.16	74	0.034*	2.04	53	0.046*
Interest	1.30	81	0.197	1.94	74	0.056	1.03	53	0.308
Understanding	0.30	81	0.766	3.19	74	0.002**	1.34	53	0.185
Learning difficulty	–2.03	81	0.045*	–2.02	74	0.047*	–2.73	53	0.009**
Engagement	1.61	81	0.112	–0.02	74	0.981	0.03	53	0.978
**Online Learning Behavior Questionnaire (OLBQ)**									
Interactive communication	–1.06	81	0.290	2.47	74	0.016*	1.93	53	0.059
Persistence	0.61	81	0.544	2.89	74	0.005**	1.23	53	0.223
Learning attitude	–0.17	81	0.869	1.79	74	0.078	2.23	53	0.030*

Sig. (2-tailed). *p < 0.05, **p < 0.01.

The responses to the four open-ended questions were examined through content analysis. Two of the authors firstly went through all the material, and then conducted two coding operations progressively: open coding and axial coding (Thorne, 2000; Bryman and Burgess, 2002). In the open coding section, a total of 142 responses were identified. In the axial coding section, connections were made for each open-ended question. The two authors worked independently so as not to influence each other. The two raters got consistent comments on most of the responses, few discrepancies were resolved through a consensual process.

## Results

### Description Results

The description information of subjects is as above (see Table 2).

#### *t*-Tests

**FIGURE 4:**
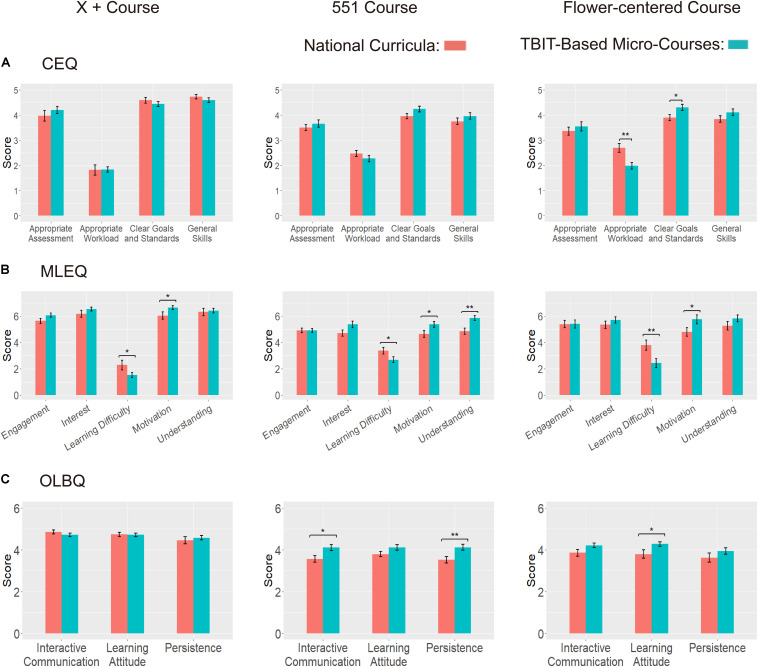
The score of the two kinds of assessment of three questionnaires on three courses. **(A)** Course Experience Questionnaire, CEQ; **(B)** Mayer Learning Experience Questionnaire, MLEQ; **(C)** Online Learning Behavior Questionnaire, OLBQ.

The independent *t*-tests results from questionnaire CEQ show that students gave equal even higher assessment on TBIT-based micro-courses on aspects “clear goals and standards,” “appropriate workload,” “appropriate assessment,” and “general skills.” Specifically, the “flower-centered course” gained higher score in “clear goals and standards” (*M* = 4.31, *SD* = 0.69) than the score of national curricula (*M* = 3.89, *SD* = 0.57; *p* = 0.019), and lower score in “appropriate workload” (*M* = 1.98, *SD* = 0.75) relative to the score of national curricula (*M* = 2.69, *SD* = 0.84; *p* = 0.002) (see Figure 4). There are no significant results on other aspects. These results partly supported the hypothesis 1 that students’ evaluation of the TBIT-based micro-courses should be better than that of national curricula (H1).

Notably, the results from MELQ showed that all the TBIT-based micro-courses got higher assessment on study motivation (*p*_home + X_ = 0.038; *p*_551_ = 0.034; *p*_Flower__–__centered_ = 0.046) and lower assessment on learning difficulty relative to the national curricula (*p*_home__+__X_ = 0.045; *p*_551_ = 0.047; *p*_Flower__–__centered_ = 0.009). Additionally, the 551-course gained higher assessment on the dimension of “understanding” (*p* = 0.002) and the marginally higher score of “interest” in MLEQ compared to the national curricula (*p* = 0.056). There are no significant differences in other dimensions. These results suggest that the TBIT-based micro-courses can better promote students’ learning motivation and reduce the learning difficulty experienced by students. To some extent, they also seemed to facilitate part of students’ understanding and possibly improve their interest in the courses (see Figure 4). These results supported Hypothesis 2 that the TBIT-based micro-courses should be more effective than the national curricula in stimulating students’ learning motivation.

Additionally, the results from questionnaire OLBQ showed that TBIT-based micro-courses gained equal even more positive assessments on aspects “interactive communication,” “persistence,” and “learning attitude” than that of national curricula. Specifically, the “551-course” gained higher scores in dimensions of “interactive communication” (*M* = 4.10, *SD* = 0.87) and “persistence” (*M* = 4.11, *SD* = 0.86) relative to the national curricula (*M* = 3.56, *SD* = 1.01, *p* = 0.016; *M* = 3.51, *SD* = 0.93, *p* = 0.005). The “flower-centered course” gained higher score on the dimension of “learning attitude” (*M*_TBIT_ = 4.27, *SD* = 0.62; *M*_national_ = 3.79, *SD* = 0.97; *p* = 0.030). There are no significant results on other dimensions. These results partly supported Hypothesis 3 that the TBIT-based micro-courses get higher assessment than national curricula.

#### Informal Interviews With Teachers

In response to the first question (i.e., the features of the courses), teachers’ answers fell under three categories: curriculum objectives, content, student learning experience. From the perspective of the teachers, the most striking feature of the courses is that the curriculum objectives focused on the cultivation of thinking abilities, real-life problem solving, and the key competencies such as autonomous development and social participation, etc. Another feature the teachers mentioned was that the courses were logically organized according to the cross-disciplinary themes or core concepts instead of being limited by the class schedule of separate subjects. Besides, they suggested that the students’ learning experience was positive. Seventy-five percentage of the teachers interviewed mentioned that the courses were interesting and could stimulate students’ motivation to learn.

In response to the second question concerning the effect of the courses on improving students’ thinking ability, more than 85% of the teachers indicated that the courses had a good effect on improving students’ thinking ability (e.g., agility and flexibility). Among them, three teachers described the specific activities which were particularly effective in improving students’ critical thinking; two teachers pointed out that the courses helped students learn how to think from multiple perspectives. Also, two teachers suggested that the effect of the curriculum on thinking ability was different from person to person and that students who are more autonomous made more progress.

The third question was about the effect on improving students’ key competencies. The answers frequently mentioned by the teachers (6 out of 14) were that the students benefited a lot from the course in autonomous development, as they reported that students’ abilities of autonomous learning and self-management have been improved. Besides, three teachers suggested that students in this course have full opportunities to practice and apply the knowledge they have learned, which is of great importance to the improvement of their competence in practical innovation. Two teachers mentioned that students’ information competence has been improved a lot through the course.

In response to the last question (i.e., students’ engagement in the courses), all teachers indicated that students showed a high willingness to participate in the learning activities. They pointed out that students in both first and fifth grade showed high levels of concentration and interest in the curriculum.

## Discussion

The present study introduces an effective instruction theory—TBIT. According to this theory, we give the specific guidance of the three critical elements of curriculum design (curriculum objectives, curriculum contents, and curriculum implementation). Next, we provide three TBIT-based micro-courses, and its instructional effects were tested. In general, the results indicated that the TBIT-based micro-courses not only improved the course quality but also enhanced students’ motivation and facilitated part of their online behaviors (such as interactive communication) of the online class. The current study has important implications for how to design effective and interesting online courses to improve students’ thinking abilities and cultivate their key competencies.

The present research indicates that TBIT has multiple beneficial effects on guiding the design of online courses, especially in terms of effective guidance for students studying at home during the period of pandemic. As for teaching objectives, every course is focused on cultivating students’ key competencies (e.g., humanistic background, scientific spirit, learning, healthy life, responsibility, and practice and innovation). Regarding teaching content, we focus on improving students’ motivation and capturing their interests, and then we designed lots of diversified, highly conceptual, interdisciplinary, and integrated contents and activities to facilitate the development of students’ thinking (e.g., flexible, critical, creative, agile, and deep thinking) and abilities (e.g., the ability of autonomous learning, independence, cooperation and communication, and follow current events, etc.). Meanwhile, according to the “learning progressions” principle (Alonzo and Steedle, 2009; Jin et al., 2019), we provided content with different difficulty levels to students in different grades to reach their zones of proximal development (Vygotsky, 1978). Regarding teaching implementation, according to the five-course design principles of TBIT (Hu et al., 2011), we set six main steps to make the class more attractive and more focused on students’ thinking and key competencies. For example, we used the information familiar to students to lead-in the course, and then set the cognitive conflict to provoking their deep thinking. Besides, we used inquiry-based teaching methods to improve multiple abilities of students, such as hand-on, independence, and interactive communication abilities, and so on. Late in the course, we usually let students summarize and reflect what they have learned in that class and try to transfer that knowledge to other situations they can be used, so as to transfer the knowledge they have learned to the abilities they have mastered and the competencies they have been cultivated. Thus, the detailed instruction leads to effective results.

Firstly, the results showed that the evaluation of TBIT-based micro-courses get equal (“appropriate assessment” and “general skills”) or even better (“clear goals and standards” and “appropriate workload”) assessments on the course quality relative to national curricula. The perception of clear goals could help students have a clear understanding about what they are going to learn, so as to improve their satisfaction with the course because a previous study has shown that students who have more clear course goals rated higher course satisfaction (Svanum and Aigner, 2011). Meanwhile, an appropriate workload in the course could avoid some potential psychological problems in students, especially in the background of the pandemic (Wang et al., 2020), as the too heavy workload is related to high test anxiety (Sansgiry and Sail, 2006). No significant results on aspects “appropriate assessment” and “general skills” mean the course designed based on TBIT could guarantee the quality of course for students as equal as the national curricular, meanwhile, cultivating the general skills for students.

Secondly, the TBIT-based micro-courses gained more assessment on positive learning experiences than that of national curricula. Specifically, TBIT-based micro-courses gained higher scores on “motivation” and lower “learning difficulty” relative to national curricula, and part of them gave a higher assessment on “understanding” and a marginally significant higher assessment on “interest” for TBIT-based micro-courses. These results reflected that students are more desired to take the course designed based on TBIT, which is consistent with the result found by Hu et al. (2016). These positive results may benefit from the design of the TBIT-based micro-courses. In this course, according to the course design principles of TBIT, in the first teaching step, teachers usually use lots of interesting information, which is closely related to the topic they plan to teach (e.g., teaching contents related to the pandemic in this study), to “lead-in” the real theme of the course (Hu et al., 2011). Meanwhile, most of the course contents are interdisciplinary. This kind of method could arouse students’ curiosity about the course, attract their attention, and engage in the course, therefore improving students’ motivation about the course (Hu et al., 2016). Notably, motivation has been consistently found to be positively related to self-control (Schmeichel et al., 2010; Vohs et al., 2012), and people who have high motivation have high autonomy and perform better in massive open online courses (MOOCs) (Durksen et al., 2016). Thus, the improvement of motivation indirectly helps solve the main problem caused by studying at home—lacking autonomous learning ability on the study (Durksen et al., 2016; Liang et al., 2020; Zhou et al., 2020). Also, the improvement of willingness, enthusiasm, and the ability for autonomous learning and self-management was evidenced by our results from teachers’ interviews.

Regarding “learning difficulty” and “understanding,” when students could actively study rely on their intrinsic motivation, they are more likely to experience lower learning difficulty about the course and have a high possibility of engaging in deeper exploration and thinking about the questions posed in that course (Hu et al., 2016; Zhang et al., 2020), so that has a better understanding of the course content.

Thirdly, students performed better in TBIT-based micro-courses in terms of online learning behavior than in national curricula. We found part of students rating higher “learning attitude,” “interactive communication,” and “persistence” on TBIT-based micro-courses than national curricula. These results suggest that students benefited from the steps of “stimulating interest and motivation,” “knowledge-construction,” and “cognitive conflict” in the TBIT-based micro-courses. Specifically, in this course, teachers usually use lots of information related to pandemic to create the situation, which is more attractive and familiar to students and can be considered as the scaffolding for students. The scaffolded content is particularly important in constructing students’ learning environments, which in turn facilitates their positive attitude toward online learning, as was found in Korhonen et al. (2018).

Regarding “interactive communication,” during thinking-based courses, teachers usually set lots of interaction steps for students to share their views, answers, and productions. Meanwhile, other teachers, parents, and students could give their opinions in the discussion section online. This process significantly facilitated the teacher-student, student-content, and student-student interactive communication in the course, which could strengthen students’ cognitive construction to relate the knowledge in their mind to the current situation, and thus facilitate their social construction with other teachers and students. As previous studies showed that the more mutual interactions between teachers and students, the more knowledge to be constructed and the more strengthened a sense of an online learning community the students will perceive (Chi, 2009; Jia et al., 2017; Ouyang et al., 2020). Meanwhile, researchers found that the high engagement in the online course can increase students’ satisfaction so that enhance their motivation to learn, thus improves students’ performance in online courses (Jin, 2017; Martin and Bolliger, 2018).

Besides, the improvement in “persistence” could be due to the step “cognitive conflict.” Because, in this process, teachers usually show information or phenomenon which were not consistent with students’ intuition or experience to provoke students’ critical thinking. Previous studies have shown that original or conflict information could attract more attention from students (Kang et al., 2010; Jia et al., 2019). Therefore, students can be more absorbed in the course and show high persistence from the “cognitive conflict” link.

In short, under the guidance of TBIT, students’ motivation for the online course has been significantly improved, the study difficulty they have experienced has deeply decreased, and their multi-thinking, -abilities, and -key competencies have been cultivated. Both the results we found from the quantity and quality data can give evidence for this conclusion. Thus, based on TBIT theory, we have solved the biggest problem that usually happened in an online course—lack self-monitoring ability (Liang et al., 2020; Shang et al., 2020)—through improving students motivation, and achieved the goal of teaching reform requirement—cultivating students’ key competencies.

There are still few limitations that need to be mentioned. Firstly, since the design of this study is not a very rigorous randomized grouping design, it might have little influence on the generalization of the results. In this study, due to the sudden outbreak of the COVID-19, we had to collect the data after the TBIT-based micro-courses have been applied. Meanwhile, since the national curricula consist of courses that students must take, TBIT micro-courses was introduced as an added-on component, rather than implemented in lieu of the traditional one. So, it was not possible, with the unexpected COVID-19 in China, for us to conduct rigorous design-based research with up and close follow-up observations every step of the way. Thus, the main aim of this study was to assess how well TBIT was received as compared to the traditional curriculum, with regard to developing students’ key competencies. In this sense, our study was meant to be a step of generating evidence for the effectiveness of TBIT, rather than a full-blown design-based study. Secondly, there might be the possibility of a Hawthorne effect in favor of TBIT. This concern was alleviated to some degree due to the following procedures. First, we didn’t introduce the TBIT-based micro-courses in a way that would bias the students in favor of this approach. Alternating between TBIT and the traditional curriculum was made seamlessly. Second, we randomly chose the classes to assess either TBIT or the traditional curriculum; this would “dilute” the possible systematic effect of making TBIT more distinct to students than the traditional counterpart. And finally, we didn’t ask students to compare their feelings about the two approaches but only asked them to report their feelings on one of them. This strategy also helped avoid the Hawthorne effect; that is, seeing one favored over the other. Another limitation is that some hypothesized advantages of TBIT are not substantiated. For example, only part of the dimensions in the questionnaires CEQ and OLBQ are improved, this might due to the time they took the experiment class was too short. It might have effects on a more wide range and the effects of the courses would be prolonger after long-time teaching. As our previous studies found that when received the courses “Learn to think,” which was designed under the guidance of TBIT, students’ abstract and concrete thinking abilities were all improved after 1 year for students in grade 1 and 2, and 6 months for grade-3-students (Hu et al., 2011). Additionally, using the same course, after a 2 years intervention, students’ scientific creativity was still increased 1 year after (Hu et al., 2013). Furthermore, after a 4 years intervention, students’ deep motivation was still increased 1 year after (Hu et al., 2016).

The current study gives lots of insights on how to design online courses in the future, extends the growing literature on the guidance role of TBIT in offline course design to the domain of online course design, and provides practical theoretical guidance for the development of online courses. Specifically, course design must focus on the key competencies of students and must be designed under the guidance of the effective teaching theory. In order to attract students’ learning motivation and interest, the online course design should reflect the interesting, comprehensive (interdisciplinary), and active nature of course content. It should enhance students’ learning interest so as to improve their motivation; it should emphasize the comprehensiveness of the content in order to reduce unnecessary learning content and decrease workload for students; it should emphasize the activation of activities so that mobilize students’ participation and interactive communication.

## Data Availability Statement

The raw data supporting the conclusions of this article will be made available by the authors, without undue reservation, to any qualified researcher.

## Ethics Statement

The studies involving human participants were reviewed and approved by the Ethics Committee of CTPAD at Shannxi Normal University. Written informed consent to participate in this study was provided by the participants’ legal guardian/next of kin.

## Author Contributions

WH, XZ, and YL designed the study. YL and XZ wrote the manuscript. XZ contributed to the data collection. YL contributed to the data analysis. WH and DD critically reviewed the manuscript. All authors read and approved the final manuscript.

## Conflict of Interest

The authors declare that the research was conducted in the absence of any commercial or financial relationships that could be construed as a potential conflict of interest.
